# Near-zero fluoroscopy pulsed field ablation using the circular-shaped catheter with ICE-guided real-time fifth electrode visualization: The FOCUS technique

**DOI:** 10.1016/j.hroo.2025.09.013

**Published:** 2025-09-19

**Authors:** Shintaro Yamagami, Toyoki Okuda, Tsukasa Motoyoshi, Masanori Murayama, Masaya Akiyama, Yuta Nakano, Hirokazu Kondo, Toshihiro Tamura

**Affiliations:** 1 Department of Cardiology, Tenri Hospital, Tenri, Nara, Japan; 2 Department of Clinical Engineering, Tenri Hospital, Tenri, Nara, Japan

**Keywords:** Atrial fibrillation, Catheter ablation, Electroanatomic mapping, Fluoroscopy reduction, Intracardiac echocardiography, Pulsed field ablation

**Key Findings**

▪ Near-zero fluoroscopy pulsed field ablation guided by intracardiac echocardiography (ICE) and 3-dimensional electroanatomic mapping using the fifth-electrode-oriented contact under SOUNDSTAR technique was feasible: the mean fluoroscopy time was 3.4 ± 2.4 seconds; 3 of 10 cases were performed with zero fluoroscopy.
▪ Continuous ICE fan alignment to the fifth electrode enabled reliable catheter–tissue contact assessment without contact-force sensing and supported complete acute pulmonary vein isolation in all 10 patients, with no complications.
▪ The workflow was reproducible under deep sedation, incorporating standardized ICE views for each pulmonary vein and over-the-wire guidance; the mean left atrial dwell time was 38.0 ± 4.9 minutes.
▪ Short-term outcomes were favorable: at 6-month follow-up, no atrial arrhythmia recurrence was documented in any patient, although longer-term durability remains to be established.

## Introduction

Pulsed field ablation (PFA) is an emerging technology for the treatment of cardiac arrhythmias. Unlike thermal ablation modalities such as radiofrequency or cryoablation, PFA delivers nonthermal energy to create myocardial lesions, enabling pulmonary vein isolation (PVI) while minimizing the risk of collateral tissue injury.^1^ The PulseSelect catheter (Medtronic, Inc), a circular PFA system, has shown similar efficacy to that of conventional thermal ablation techniques in achieving durable PVI, with a favorable safety profile.^2^ However, this system lacks integrated tools to assess real-time tissue contact. Consequently, fluoroscopy is typically required to confirm catheter-tissue apposition, raising concerns about radiation exposure for patients and operators. The fifth electrode, angled 20° anteriorly, is critical for ensuring myocardial contact and is the most easily visualized under fluoroscopy. We report a novel technique that achieves durable PFA with minimal fluoroscopy using intracardiac echocardiography (ICE) to visualize the fifth PulseSelect catheter electrode in real time and confirm tissue contact.

## Case report

This case series included 10 consecutive patients who underwent initial catheter ablation for atrial fibrillation (AF) with the PulseSelect catheter (8 persistent, 2 paroxysmal). The mean age was 67.6 ± 9.4 years; 6 were male. Preprocedural computed tomography was used to evaluate left atrial (LA) and pulmonary vein (PV) anatomy, identify anatomic variations, and enhance the accuracy of 3-dimensional (3D) mapping integration.

2 procedures were performed under general anesthesia, whereas the remaining 8 were conducted under deep sedation using propofol. AF ablation was performed with near-zero fluoroscopy in all 10 cases using the CARTO 3 system (Biosense Webster) and the method outlined below. The research reported in this paper adhered to the principles outlined in the Declaration of Helsinki.

**Figure 1:**
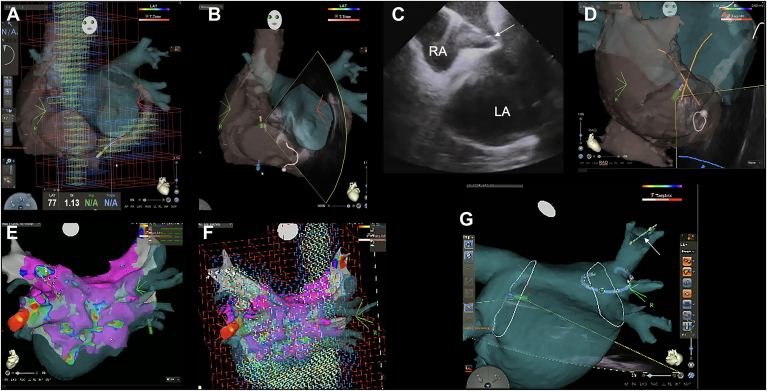
CARTO-guided workflow for near-zero fluoroscopy PFA using the PulseSelect catheter. **A:** Matrix of the RA, SVC, IVC, and CS, constructed using an OCTARAY catheter without fluoroscopy. **B:** CARTO 3D image showing the SOUNDSTAR catheter during transseptal puncture. The ultrasound fan is displayed to assist in confirming catheter orientation and safe passage through the septum. **C:** ICE image during transseptal puncture using an ACCUSAFE needle (*white arrow*). **D:** CARTO 3D visualization of the interatrial septum. A cross (“×”) mark indicates the previously confirmed puncture site, and the SOUNDSTAR catheter tip is directed toward it and advanced into the LA. **E:** LA anatomic map acquired using an OCTARAY catheter. **F:** Same LA anatomy as in panel E, shown with matrix display enabled to confirm adequate mapping coverage prior to ablation. **G:** Visualization of the wire tip during PFA. The wire is connected with an alligator clip to render its position visible on CARTO; the tip (*white arrow*) is advanced into the RSPV under 3D guidance. CS = coronary sinus; ICE = intracardiac echocardiography; IVC = inferior vena cava; LA = left atrium; PFA = pulsed field ablation; RA = right atrium; RSPV = right superior pulmonary vein; SVC = superior vena cava; 3D = 3-dimensional.

Vascular access was obtained under ultrasound guidance, and heparin was used to maintain an activated clotting time goal of 300–350 seconds. An OCTARAY catheter (Biosense Webster) was first used to map the right-sided structures, including the right atrium, superior vena cava, inferior vena cava, and coronary sinus, creating a 3D matrix that was then merged with preacquired computed tomography images (Figure 1A). After completion of the right-sided matrix acquisition, a transseptal puncture was performed using an ACCUSAFE transseptal needle (Baylis Medical) under ICE catheter (SOUNDSTAR, Biosense Webster) guidance. Subsequently, a FlexCath Advance sheath (Medtronic) was inserted directly into the LA (Figure 1B and 1C). The SOUNDSTAR catheter was then introduced into the LA through the same transseptal site. For guidance, the puncture site was marked as a × symbol on the CARTO map, allowing the SOUNDSTAR catheter to be advanced into the LA under 3D navigation (Figure 1D).

We created a matrix and preablation voltage map of the LA, PVs, and LA appendage using the OCTARAY catheter, confirmed that sufficient data were acquired for the LA and each PV (Figure 1E and 1F), and then advanced the PulseSelect catheter into the LA for ablation. To enable fluoroscopy-free PV selection, we attached an alligator clip to the guidewire’s proximal end so its distal tip could be visualized on the 3D mapping system (Figure 1G). We then selected the target PV and advanced the PulseSelect catheter over the wire, positioning it at the ostium and antrum of each of the 4 PVs.

**Figure 2:**
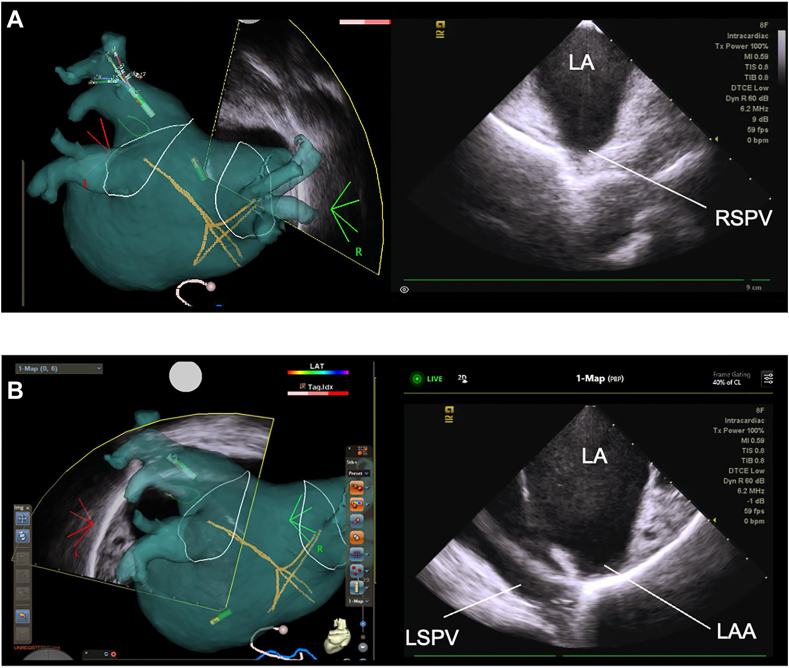
ICE catheter positioning for PV visualization during PFA. **A:** During right PV isolation, the ICE catheter tip was positioned centrally within the LA. By tilting the catheter anteriorly and inferiorly, the RSPV and RIPV were visualized. **B:** During left PV isolation, the ICE catheter tip was advanced further leftward. Posterior deflection enabled visualization of the left PVs from an inferior angle. Additional leftward and rightward deflection allowed clear visualization of the LSPV and LIPV, respectively. ICE = intracardiac echocardiography; LA = left atrium; LAA = left atrial appendage; LIPV = left inferior pulmonary vein; LSPV = left superior pulmonary vein; PFA = pulsed field ablation; PV = pulmonary vein; RIPV = right inferior pulmonary vein; RSPV = right superior pulmonary vein.

The basic ICE positioning during PVI was standardized as follows. The ultrasound fan was consistently displayed on the 3D mapping system and served as a reference to guide real-time catheter manipulation. During isolation of the right PVs, the ICE catheter tip was positioned centrally within the LA. By tilting the catheter anteriorly and inferiorly, the right superior PV and right inferior PV were visualized (Figure 2A). For isolation of the left PVs, the ICE catheter tip was advanced further leftward compared with the position used for right PVI. By deflecting the tip posteriorly, the left PVs were visualized from an inferior viewpoint. Additional leftward and rightward deflection allowed for optimal visualization of the left superior PV and left inferior PV, respectively (Figure 2B).

**Figure 3:**
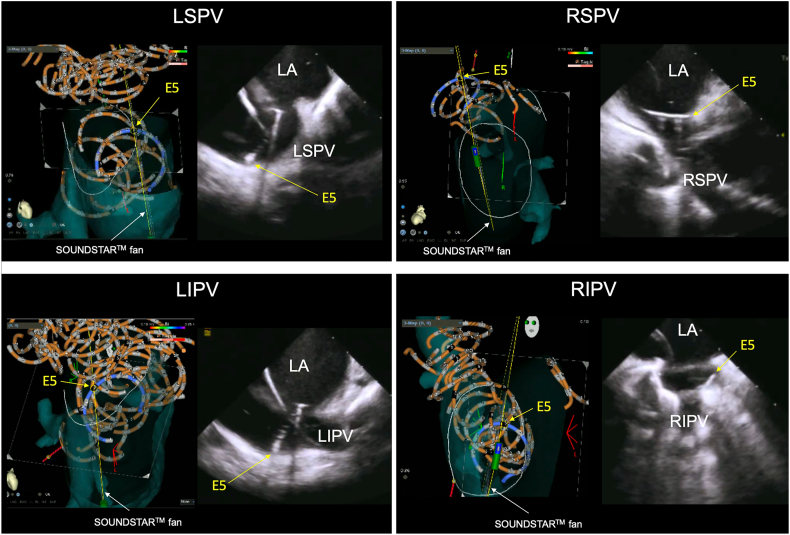
ICE-guided PulseSelect PFA using the FOCUS technique in each PV. Panels demonstrate the 3D CARTO view combined with ICE for each PV during PFA. The fifth electrode of the PulseSelect catheter is positioned toward the target region under ICE guidance with the SOUNDSTAR fan aligned to confirm myocardial contact. This technique allows real-time verification of contact between the fifth electrode and the myocardium during PFA, contributing to safe and effective lesion delivery in a near-zero fluoroscopy workflow. E5 indicates the fifth electrode. FOCUS = fifth-electrode-oriented contact under SOUNDSTAR; ICE = intracardiac echocardiography; LA = left atrium; LIPV = left inferior pulmonary vein; LSPV = left superior pulmonary vein; PFA = pulsed field ablation; PV = pulmonary vein; RIPV = right inferior pulmonary vein; RSPV = right superior pulmonary vein; 3D = 3-dimensional.

Regarding the PVI procedure, a minimum of 4 applications was delivered at the ostium and antrum of each of the 4 PVs. During each of the 4 applications, the catheter was repositioned in the superior, inferior, left, or right direction, with the no. 5 electrode oriented accordingly to achieve full circumferential isolation of the PV. Throughout the entire process, the ultrasound fan of the ICE catheter was consistently directed toward the no. 5 electrode in real time. To ensure that the ultrasound beam was consistently targeting the no. 5 electrode in real time, the 3D mapping view was carefully adjusted so that the ICE fan appeared as a tangential line across the no. 5 electrode. This allowed continuous confirmation that the electrode was within the imaging plane of the ultrasound beam (Figure 3). We refer to this workflow as the fifth-electrode-oriented contact under SOUNDSTAR (FOCUS) technique. This visualization confirmed that the electrode was precisely within the ultrasound beam path, allowing continuous confirmation of effective myocardial contact at the target site. A procedural recording of PFA applied to the right superior PV using the FOCUS technique is shown in Supplemental Video 1. All applications were delivered only after adequate contact had been verified and complete PVI was confirmed by entrance block testing using the OCTARAY catheter. Throughout the entire procedure, fluoroscopy was minimized and restricted to safety-critical steps—specifically, brief confirmation during transseptal puncture and, when guidance by 3D mapping with ICE alone was insufficient, during advancement of the SOUNDSTAR catheter into the LA across the transseptal site.

Complete PVI was achieved in all 10 cases without complications, and no further applications were needed after the initial circumferential ablation around each PV. 2 subjects underwent posterior wall isolation, during which pacing with the OCTARAY catheter demonstrated entrance and exit block. An average of 47.3 ± 9.2 applications were delivered. Of the 10 cases, 3 were performed with zero fluoroscopy, whereas the remaining 7 required brief fluoroscopy (all ≤10 seconds). The mean fluoroscopy and LA dwell times were 3.4 ± 2.4 seconds and 38.0 ± 4.9 minutes, respectively. During the 6-month follow-up, no atrial arrhythmia recurrence was documented in any patient. Although thermal ablation techniques have been successfully used for many years, their application is limited by the risk of collateral tissue damage.^3^ Conversely, PFA creates myocardial lesions through nonthermal mechanisms, thereby avoiding injury to surrounding structures.^2^ The PULSED AF trial previously demonstrated that the PulseSelect catheter can effectively treat AF without causing thermally mediated complications.^2^ However, one limitation of this catheter is the absence of objective feedback parameters, such as contact force measurement, to confirm adequate tissue–catheter contact. Given the established correlation between lesion depth and electrode–tissue proximity,^4^ insufficient contact during ablation may compromise lesion formation. Therefore, when using the PulseSelect catheter, additional strategies are required to ensure optimal tissue contact and effective energy delivery. The positioning of the circular array of the PFA catheter is generally guided by fluoroscopy, ICE, or 3D mapping systems. Fluoroscopy is the most standard method for confirming catheter-to-myocardium contact. However, frequent use of fluoroscopy increases radiation exposure for patients and operators. In fact, in PFA catheters lacking contact force sensing, fluoroscopy time and radiation dose have been reported to be higher than thermal ablation.^5^ Importantly, 3D mapping systems are also helpful in recognizing the location and alignment of the catheters. However, skeletal muscle twitching and/or dry cough, which PFA frequently causes, may result in patient body movement and shifting of the anatomic model within the mapping system. Therefore, particularly when the procedure is performed under deep sedation, it may be preferable to supplement 3D mapping with intermittent fluoroscopic confirmation, rather than relying solely on the 3D mapping system for ablation guidance. Furthermore, although ICE is undoubtedly a valuable tool for assessing tissue–catheter contact, it is often difficult to precisely identify which specific electrode is being visualized on the imaging plane. This limitation is particularly relevant when using catheters like the PulseSelect, where lesion formation depends on ensuring adequate contact at a specific electrode. Without precise electrode localization, there remains a risk that the intended electrode may not be in effective contact with the myocardium, despite appearing well positioned on ICE.

The FOCUS technique overcomes ICE’s limitation and is expected to enhance PulseSelect PFA effectiveness under ICE guidance. Aligning the SOUNDSTAR fan with the fifth electrode—PulseSelect’s focal energy–delivery point—confirms precise, real-time catheter–myocardial contact. Thus, FOCUS may improve lesion durability with the PulseSelect catheter.

Although the clinical utility of fluoroscopy-reducing or fluoroscopy-free PFA techniques has been increasingly reported,^6^ many of these approaches have been performed under general anesthesia. Conversely, the FOCUS technique offers a significant advantage in procedures performed under deep sedation, where coughing or body movement can cause displacement of the 3D anatomic map. By enabling real-time visualization of myocardial contact at a specific electrode without the need for fluoroscopy, the FOCUS technique allows for effective and safe PulseSelect PFA even under deep sedation.

In all 10 patients, including most under deep sedation, postablation mapping showed durable isolation with no residual PV potentials or need for additional applications, suggesting that real-time ICE visualization of the fifth electrode may enable more targeted lesion delivery.

This study has several limitations. First, the sample size was limited to 10 consecutive patients at a single center. Second, long-term follow-up data are unavailable, so lesion durability in the chronic phase remains unconfirmed. Third, although the fifth electrode served as a surrogate for tissue contact, its quantitative relationship with lesion quality is unvalidated. Fourth, requiring integration-capable electroanatomic mapping and ICE systems may limit generalizability in resource-limited settings. Fifth, we did not undertake a formal comparison of procedure duration with fluoroscopy-guided cases because our center transitioned from fluoroscopy-guided PFA to a near-zero fluoroscopy workflow during the study period, introducing time-dependent confounding (eg, learning-curve effects).

Nonetheless, this case series demonstrates that PFA with the PulseSelect catheter can be performed under near-zero fluoroscopy. Integrating CARTO 3D mapping and ICE-guided real-time contact confirmation enables safe, precise PVI without fluoroscopy. This reproducible strategy may enhance safety and consistency across clinical settings, although further studies should assess its efficacy and feasibility.

## Disclosures

The authors have no conflicts of interest to disclose.

## References

[1] Verma A., Boersma L., Haines D.E. (2022). First-in-human experience and acute procedural outcomes using a novel pulsed field ablation system: the PULSED AF pilot trial. Circ Arrhythm Electrophysiol.

[2] Verma A., Haines D.E., Boersma L.V. (2023). Pulsed field ablation for the treatment of atrial fibrillation: PULSED AF pivotal trial. Circulation.

[3] Andrade J.G., Champagne J., Dubuc M. (2019). Cryoballoon or radiofrequency ablation for atrial fibrillation assessed by continuous monitoring: a randomized clinical trial. Circulation.

[4] Howard B., Verma A., Tzou W.S. (2022). Effects of electrode-tissue proximity on cardiac lesion formation using pulsed field ablation. Circ Arrhythm Electrophysiol.

[5] Aldaas O.M., Malladi C., Han F.T. (2024). Pulsed field ablation versus thermal energy ablation for atrial fibrillation: a systematic review and meta-analysis of procedural efficiency, safety, and efficacy. J Interv Card Electrophysiol.

[6] Bulava A., Hanis J., Eisenberger M. (2015). Catheter ablation of atrial fibrillation using zero-fluoroscopy technique: a randomized trial. Pacing Clin Electrophysiol.

