# Supplementary material for: Near-zero fluoroscopy pulsed field ablation using the circular-shaped catheter with ICE-guided real-time fifth electrode visualization: The FOCUS technique
Source: Heart Rhythm O2. 2025 Sep 19;6(12):2011–5. doi: 10.1016/j.hroo.2025.09.013 (PMC12800800; doi:10.1016/j.hroo.2025.09.013)
Supplement: Supplementary Video Legend [file mmc2.docx]

**Video 1. PFA at the RSPV with the FOCUS technique**
This clip demonstrates near-zero fluoroscopy PFA at the anterior wall of the RSPV. The 5th electrode of the PulseSelect^TM^ catheter is clearly highlighted on the CARTO 3D system and visualized in real time with the SOUNDSTAR^TM^ catheter. The SOUNDSTAR^TM^ fan is intentionally aligned with the 5th electrode to confirm tissue contact prior to ablation. PFA, pulsed field ablation; RSPV, right superior pulmonary vein.
